# Limitations of broadly trained LLMs in interpreting orthopedic Walch glenoid classifications

**DOI:** 10.3389/frai.2025.1644093

**Published:** 2025-08-28

**Authors:** Adam ElSayed, Gary F. Updegrove

**Affiliations:** Penn State Health Milton S. Hershey Medical Center, Hershey, PA, United States

**Keywords:** Claude 3.5-sonnet, orthopaedic surgery, DeepSeek R1, Walch glenoid morphology, large language model, shoulder osteoarthritis, Walch glenoid type

## Abstract

Artificial intelligence (AI) integration in medical practice has grown substantially, with physician use nearly doubling from 38% in 2023 to 68% in 2024. Recent advances in large language models (LLMs) include multimodal inputs, showing potential for medical image interpretation and clinical software integrations. This study evaluated the accuracy of two popular LLMs, Claude 3.5 Sonnet and DeepSeek R1, in interpreting glenoid diagrams using Walch glenoid classification in preoperative shoulder reconstruction applications. Test images included seven black-white Walch glenoid diagrams from Radiopedia. LLMs were accessed via Perplexity.ai without specialized medical training. LLMs were tested across multiple conversation threads with prompt instructions of varying length, ranging from 22 to 864 words for DeepSeek and 127 to 840 words for Claude. Performance differed significantly between models. DeepSeek achieved 44% accuracy (7/16), while Claude had 0% accuracy (0/16). DeepSeek showed a mild positive correlation between instruction length and response accuracy. Common errors across both LLMs included misclassifying A2 as either A1 (32%) or B2 (20%). Results highlight limitations in broadly trained LLMs’ ability to interpret even simplified medical diagrams. DeepSeek’s continuous learning feature and open-source dataset integration exhibited superior accuracy, although it was still insufficient for clinical applications. These limitations stem from LLM training data containing primarily text instead of medical images, creating pattern recognition deficiencies when interpreting visual medical information. Despite AI’s growing adoption in healthcare, this study concludes that as of February 2025, publicly available broadly trained LLMs lack the consistency and accuracy necessary for reliable medical image interpretation, emphasizing the need for specialized training before clinical implementation.

## Introduction

Artificial intelligence (AI) is an inevitable evolution to digital workflows, with online large language model (LLM) chatbot, such as ChatGPT, significantly increasing AI’s accessibility and usage across fields such as computer science, customer service, and even medicine. AMA’s 2025 Augmented Intelligence Research Survey reveals that physicians’ use of AI in clinical settings has nearly doubled from 38% in 2023 to 68% in 2024 (AMA, 2025), which highlights AI’s rapidly growing impact on physician workflows. This growth is likely to continue given the rapid evolution of LLMs; March 2023 marked the release of GPT 4.0 and its innovations to multimodal input, which allows LLMs to interpret image and text inputs simultaneously (Thirunavukarasu et al., 2023). Since then, ChatGPT competitors, such as Claude 3.5 Sonnet, are proving themselves more accurate than ChatGPT 4.0 at diagnosing acute ischemic stroke from medical imaging (Koyun and Taskent, 2025), and most recently, DeepSeek’s groundbreaking development of a free-to-use and open-source reasoning model, R1, has opened new possibilities for AI integrations in clinical software (Temsah et al., 2025). Newer studies similarly recognize superior diagnostic capabilities from Claude 3.5 Sonnet and DeepSeek R1 compared to ChatGPT 4.0, identifying them both as enhancements to disease classification and clinical decision-making (Kurokawa et al., 2024; Gupta and Pande, 2025).

Orthopedic surgery and radiology have been particularly invested in LLMs, with ChatGPT 4.0 being regarded as a powerful tool with the potential to improve accuracy, efficiency, cost of care, and patient outcomes due to decreased delays in diagnosis (Srivastav et al., 2023). In shoulder surgery, Walch glenoid classification is the most widely used assessment of glenoid morphology and wear in preparation for shoulder reconstruction procedures. This classification was based on axillary shoulder radiographs and axial computed tomography (CT) images to evaluate glenoid erosion patterns in primary glenohumeral osteoarthritis (Zimmer et al., 2020).

The Walch glenoid classification has evolved over time, with recent updates, including glenoid types A, B, C, and D. Type A features centered humeral heads with concentric wear and no subluxation, classified as A1 if a line from anterior to posterior glenoid rim does not transect the humeral head, and A2 if it does (Bercik et al., 2016; Barnsley et al., 2025). Type B involves asymmetric wear with posterior subluxation: B1 has only posterior joint space narrowing and B2 shows biconcave humeral head with posterior rim-erosion and retroversion <15°, while B3 exhibits monoconcave humeral head with posterior wear and retroversion >15° and/or >70% posterior subluxation (Bercik et al., 2016; Barnsley et al., 2025). Type C presents dysplastic humeral heads with retroversion >25° not from erosion (Bercik et al., 2016; Barnsley et al., 2025). Type D displays anteversion and/or anterior subluxation <40% regardless of concavity (Bercik et al., 2016; Barnsley et al., 2025).

Despite the widespread adoption of the Walch classification system, studies have consistently reported inconsistent reliability among orthopedic surgeons (Schaefer et al., 2024). While recent research has demonstrated the potential of deep learning models for Samilson–Prieto glenohumeral osteoarthritis classification on radiographs (Magnéli et al., 2024), there remains a significant gap in evaluating LLMs specifically for glenoid morphology classification. Given the importance of classifying glenoid wear, combined with the lack of existing literature surrounding potential AI use cases, this initial study aims to explore the applications of publicly accessible and broadly trained LLMs in utilizing Walch glenoid classification to distinguish images of glenoid diagrams. Considering the disproportionately low amounts of research on non-ChatGPT LLMs, this study focuses on comparing the accuracy of Claude 3.5 Sonnet and DeepSeek R1 in Walch glenoid classifications, in addition to analyzing common mistakes and correlations between accuracy and prompt wordcount.

## Methods

**Figure 1:**
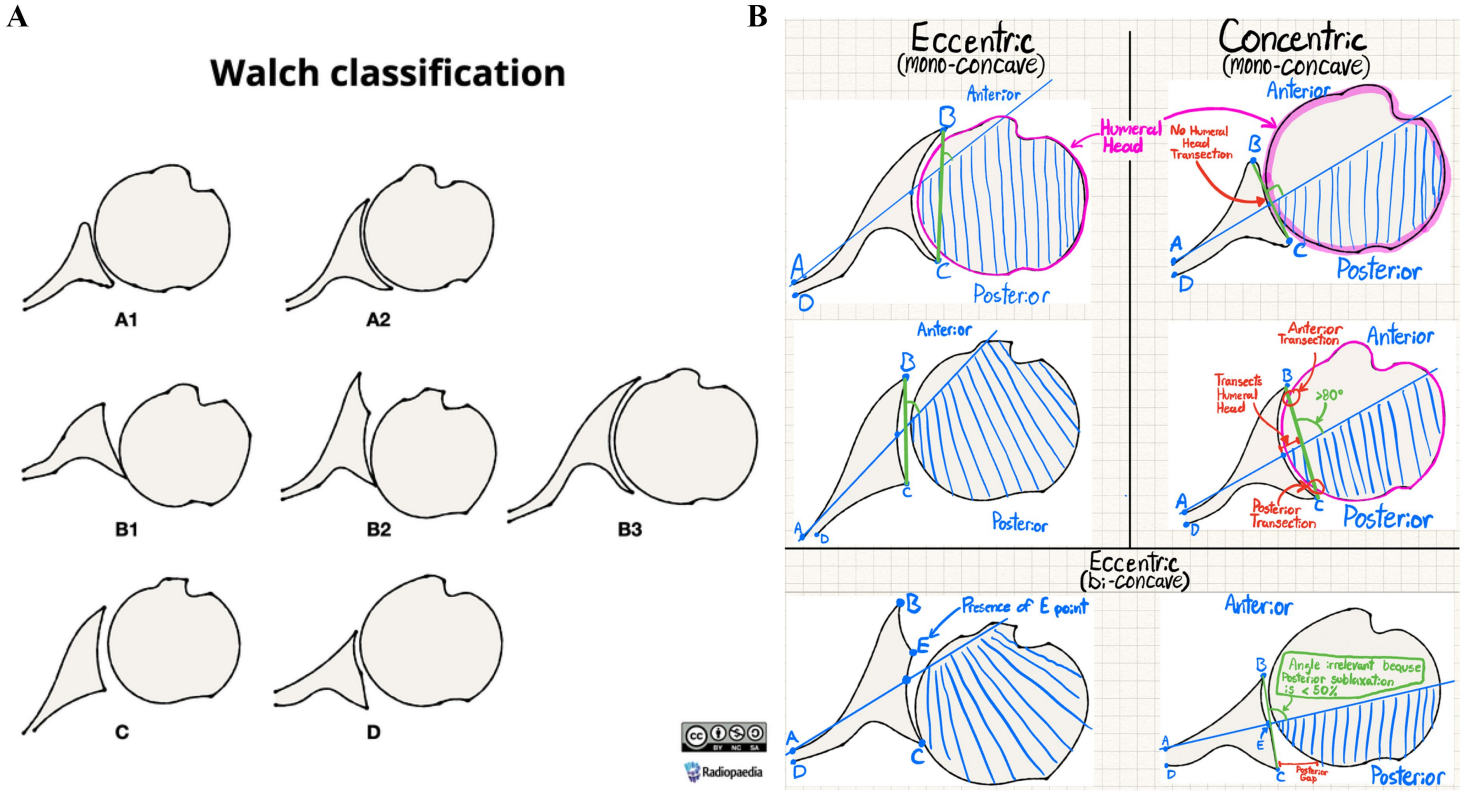
**(A)** Radiopedia’s Walch glenoid classification reference image (reproduced with permission from Knipe (2025) following approval from https://radiopaedia.org/ (ID:202513-3867), licensed under CC-BY-SA). **(B)** Annotated version of Radiopedia’s Walch glenoid classification diagram for AI use.

The seven images used for this study were obtained from Radiopedia (Knipe, 2025), with each image illustrating a black-white diagram corresponding with each Walch type (Figure 1A). Additional figures were not deemed necessary due to the seven images’ coverage of all Walch glenoid types, including A1, A2, B1, B2, B3, C, and D. An 87.5% of queries (28/32) analyzed accuracy in interpreting glenoid types A1, A2, B1, and B2 reflecting their combined 91% prevalence among primary glenohumeral arthritis cases (Zimmer et al., 2020). The AI models utilized in this study, Claude 3.5 Sonnet and DeepSeek R1, were accessed via Perplexity—a publicly accessible website where “Pro” subscription users can switch between popular LLMs such as ChatGPT and Claude, and recently the addition of DeepSeek R1. LLMs used were default models without specialized medical training.

Various conversation threads were opened with both Claude 3.5 Sonnet and DeepSeek R1, with the initial prompt containing instructions of varying length detailing how to use the Walch glenoid classification to interpret future diagrams. All prompts were uploaded alongside Radiopedia’s Walch glenoid classification with illustrative annotations distinguishing each glenoid’s visual characteristics (Figure 1B), although prompts varied in word count. No additional context was provided for queries aside from the instructions. Examples of prompts can be found in the supplementary materials.

After receiving acknowledgment of the initial instructions, most threads were asked follow-up queries to test their use of prior instructions in classifying an attached screenshot of one of the seven Radiopedia Walch glenoid diagrams. To better analyze the accuracy of the initial prompt, two threads were prompted to classify a Walch glenoid diagram in the same initial query that contained instructions.

After each LLM provided its response, metrics of each response were collected in a spreadsheet, including each prompt, prompt wordcount, AI responses, LLM name, correct Walch glenoid classification, and AI’s Walch glenoid classification. LLM performance was evaluated by comparing the accuracy of the AI’s classification with the correct Walch glenoid classification for a given prompt. Prompt wordcount was collected to analyze correlations between the length of prompts and the accuracy of AI outputs.

## Results

A total of seven conversation threads with 16 queries running DeepSeek R1 were compared to a total of 10 conversation threads with 16 queries running Claude 3.5 Sonnet, which was adjusted to remove any AI responses that were neither correct nor incorrect (5 DeepSeek R1 and 18 Claude 3.5 Sonnet) due to being acknowledgments of instructions.

**Figure 2:**
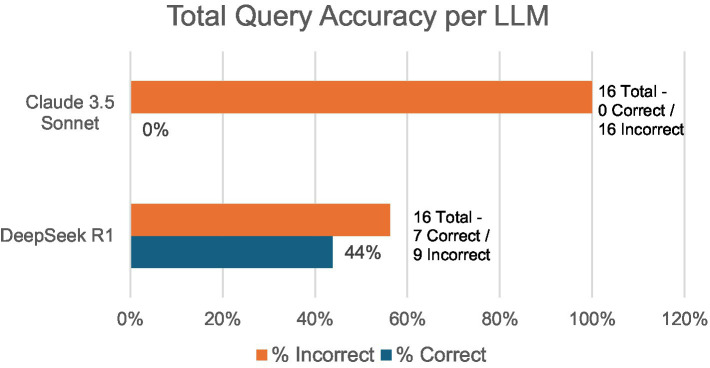
Total accurate responses/total responses per LLM.

In total, DeepSeek R1 saw 44% total accuracy (7/16) compared to Claude Sonnet’s 0% (0/16) total accuracy (Figure 2). For DeepSeek R1, the number of follow-up queries ranged from 0 to 6, while Claude 3.5 Sonnet ranged from 1 to 3. Threads with 0 follow-ups represent two conversation threads where initial instructions were combined with a diagram to be interpreted. No other queries combined initial instructions with a Walch glenoid classification test.

**Figure 3:**
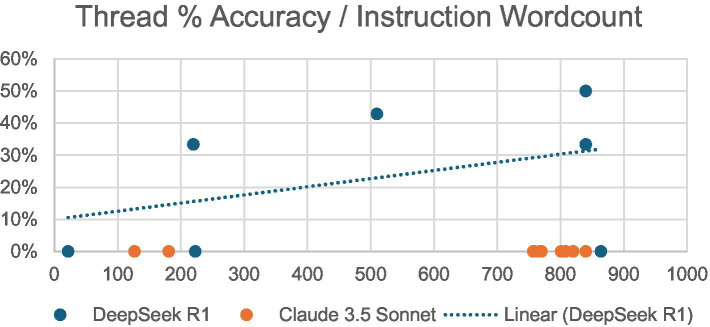
Percentage of accurate responses/wordcount of initial prompt per LLM.

Additionally, the relationship was observed between the word count of a conversation thread’s initial prompt and the accuracy of the AI’s future responses within that thread (Figure 3). Initial instruction word counts ranged from 22 to 864 for DeepSeek R1 and from 127 to 840 for Claude 3.5 Sonnet. DeepSeek R1 demonstrated a mildly positive correlation, with the most accurate thread (50%) having the second-highest word count (840). Claude 3.5 Sonnet yielded no correct responses regardless of word count.

**Figure 4:**
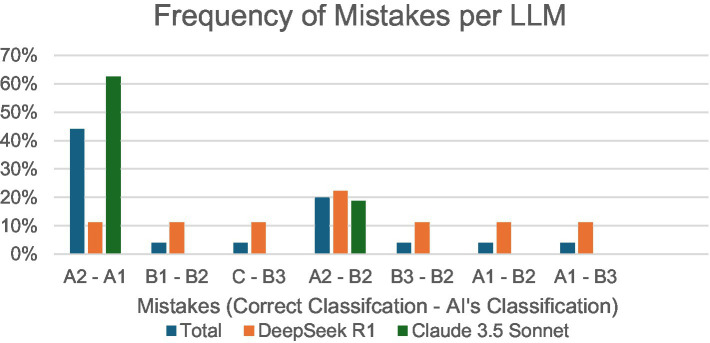
Percentage of specific incorrect input–output combinations/total incorrect responses, per LLM.

Finally, incorrect answers were analyzed to observe the most frequent mistakes made by each LLM (Figure 4). In total, the most common mistake across both LLMs (32%) was classifying A2 as A1, with 20% of mistakes being from classifying A2 as B2. DeepSeek R1’s most common mistake (22%) was classifying A2 as B2, with all other mistakes tied at 11% frequency. Claude 3.5 Sonnet’s most common mistake (63%) was classifying A2 as A1, followed by classifying A2 as B2 (19%) or B3 (13%), respectively.

## Discussion

This study was originally supposed to observe the accuracy of publicly available LLMs in using Walch glenoid classification to classify deidentified computed tomography (CT) glenoid images. However, the AI’s inconsistencies and inaccuracies with Radiopedia’s straightforward and high-contrast classification diagrams (Knipe, 2025) proved that moderate image interpretation skills, such as consistent and accurate identification of curves and spaces, are beyond the capabilities of today’s publicly accessible, broadly trained LLMs. Thus, given their shortcomings in these simple tasks, we concluded neither Claude 3.5 Sonnet nor DeepSeek R1 can reliably reproduce accurate analysis of diagrams, let alone real clinical imaging such as CT scans, in the context of Walch glenoid classifications.

Mid-January 2025 marked the release of DeepSeek R1, which, in this study, demonstrated significantly increased performance compared to Claude 3.5 Sonnet. Analysis of mistake frequencies shown in Figure 4 highlights DeepSeek R1’s superior attention to detail in multimodal medical image interpretation, including wear patterns and humeral head positioning, compared to Claude 3.5 Sonnet. While Claude 3.5 Sonnet exhibits a pronounced vulnerability to A2-A1 misclassification (63%), representing the critical distinction between centered and decentered glenoids, DeepSeek R1 maintains relatively uniform error rates (0–22%) across all classification pairs. Recent comparative studies reveal that while LLMs, such as Claude 3.5 Sonnet, may excel in language-driven or broad-context tasks, their overconfidence despite incorrect outputs for certain complicated tasks, including multimodal clinical diagnoses, causes disproportionately clustered error patterns (Suh et al., 2024) similar to Claude 3.5’s error distribution pattern in this study. These findings underscore the importance of evaluating both overall accuracy and the distribution of errors, as models with more consistent performance profiles, such as DeepSeek R1, may offer greater clinical utility and safety (Ahmed et al., 2025).

This innovative “reasoning” model outperforms other LLMs due to its continuous learning feature, entailing ongoing automatic integration of publicly available open-source datasets in its training data (Temsah et al., 2025), with potential inclusion of medical illustration data from sources such as Radiopedia or other Creative Commons licensed repositories. DeepSeek R1’s open-source code also uniquely empowers a global community of researchers and developers to collaboratively improve and customize its capabilities for specific use cases, including clinical image interpretation (Temsah et al., 2025). Moreover, another study identified DeepSeek R1 as more comprehensive and readable when discussing orthopedic surgical procedures compared to other LLMs (Zhou et al., 2025). Although speculative, these characteristics offer plausible explanations for DeepSeek R1’s superior results. Nonetheless, DeepSeek’s performance is only impressive when compared to other LLMs, with the study concluding that DeepSeek R1’s outputs are “fair” under the DISCERN criteria despite being the best, highlighting the needs for improvement and personalization (Zhou et al., 2025).

For all LLMs, including Claude 3.5 Sonnet and DeepSeek R1, AI performance is significantly limited by the type of data it is trained on, which directly influences an LLM’s ability to recognize patterns and synthesize multimodal information (Srivastav et al., 2023). Traditionally, publicly accessible LLMs are broadly trained using text data, such as websites, social media, and books, to predict word sequence patterns to create responses to prompts (Parillo et al., 2024); out of these LLMs, none of them have had exposure to medical data, such as patient records, lab data, or medical imaging (Meskó and Topol, 2023). This lack of medical data has been directly correlated with decreased performance in ChatGPT, particularly when exposed to novel image types, conditions, or patient populations (Srivastav et al., 2023).

Other studies analyzing broadly trained LLMs also report similar findings. One analysis of ChatGPT-4 V’s accuracy in answering electrocardiogram multiple-choice questions found the AI particularly weak at reading visual parameters, such as PR intervals (Zhu et al., 2024). Another study using a broadly trained GPT-4 model for mammographic interpretation reported a high frequency of hallucinations and concluded that future clinical applications of LLMs require rigorous training and validation to be considered reliable (Pesapane et al., 2025).

Risks of untrained AI in medical applications include “hallucination” responses that are confident despite being incorrect, if not made-up, which can have drastic effects if used to influence patient care (Meskó and Topol, 2023). Broadly trained LLMs are at particular risk of hallucinations due to training data often including misinformation and biases (Parillo et al., 2024). Furthermore, accuracy is further impaired by AI’s lack of access to electronic medical records, which prevents the formulation of case-specific answers (Parillo et al., 2024). Considering Walch glenoid classification’s role in surgical planning and implant selection, AI misclassifications carry significant risk for impacting patient-survival rates by increasing revision rates, decreasing prosthetic longevity, and decreasing functional outcomes (Vo et al., 2017).

### Limitations

This study assessed a limited cohort (*n* = 16), which restricts the generalizability of the findings, including the mildly positive correlation between prompt wordcount and DeepSeek’s performance. Additionally, there was disproportionate analysis across various glenoid types, with 76% (24/32) of queries testing analysis of type A1 or A2 glenoids. AI training utilized only seven Radiopedia diagrams and selected peer-reviewed sources, which were all provided simultaneously, potentially increasing hallucination frequency compared to longitudinal exposure to a larger quantity of literature and reference images. Variability in prompt wording and length between trials introduced confounding output differences. Additionally, differences in conversation thread length complicate longitudinal comparisons. Finally, LLM access through Perplexity.ai, instead of their native platforms, risks potential platform-specific performance biases.

Future investigations on orthopedic applications of multimodal AI LLMs should include larger sample sizes, utilization of consistent prompts between conversation threads, and emphasis on clinical applicability by analyzing different LLMs, including models pre-trained on clinical datasets that include annotated radiographs and CT scans.

## Conclusion

Although AI integrations show promise to benefit both patient care and provider workflows, especially with exponentially evolving capabilities of newer reasoning models, maximizing the potential of LLMs requires extensive database training to provide outputs that are case-specific and medically accurate (Parillo et al., 2024). In conclusion, distinguishing the potential of AI in improving healthcare workflows from its current capabilities is extremely important, particularly given its increasing use among providers. In the context of image interpretation, our study demonstrated that publicly available and broadly trained LLMs as of February 2025 did not have the ability to consistently and accurately recognize and interpret Walch glenoid classification diagrams, let alone radiographic images.

## Data Availability

The raw data supporting the conclusions of this article will be made available by the authors, without undue reservation.
