# Supplementary material for: Limitations of broadly trained LLMs in interpreting orthopedic Walch glenoid classifications
Source: Front Artif Intell. 2025 Aug 28;8:1644093. doi: 10.3389/frai.2025.1644093 (PMC12424433; doi:10.3389/frai.2025.1644093)
Supplement: Supplementary file 1 [file Data_Sheet_1.docx]

Supplementary Materials – Prompt Examples

Example 1:

Instructions for Glenoid Image Analysis:

Please use the attached reference diagrams to visually interpret glenoid images. Follow the steps below to classify the glenoid type accurately.

Step 1: Construct the Scapular Line

Purpose: Establish a reference line for measuring humeral head subluxation and glenoid version.

Procedure: Identify Point A (medial scapular border) and draw a straight line through the midpoint of the glenoid (between Points B and C) extending laterally.

The midpoint should represent the geometric center of the glenoid and must not lie directly on the B-C line.

Extend this line laterally through the humeral head to assess posterior subluxation (highlighted in blue in the diagrams).

Use this line as the baseline for subsequent measurements.

Step 2: Evaluate Humeral Head Subluxation

Purpose: Determine whether posterior subluxation is <50% or >50% to differentiate Type D glenoids.

Criteria: If posterior subluxation is <50%, confirm: There is a disproportionately larger gap between Point C (posterior rim) and the humeral head compared to Point B (anterior rim).

If both conditions are true, classify as Type D.

If posterior subluxation is ≥50%, proceed to Step 3.

Step 3: Assess Glenoid Concentricity

Purpose: Differentiate concentric from eccentric glenoids, a critical step in classification.

Procedure: Draw the B-C line (anterior-to-posterior rim, shown in green). Measure the acute angle where this green line intersects the scapular line (blue).

Thresholds:

Angle >75° = Concentric

Angle <75° = Eccentric

Step 4: Classify Glenoid Type

A. Concentric Glenoids

Associated only with Type A classification.

Type A1:

Minor central wear or erosion.

The B-C line does not transect or is tangential to the humeral head surface.

Example: Top-right image in reference diagram.

Type A2:

Severe central wear or erosion.

The B-C line transects the humeral head at both anterior and posterior points (as shown in red in bottom-right image of reference diagram).

Confirm by identifying both anterior and posterior transection points.

B. Eccentric Glenoids

Associated with Types B, C, or D classifications.

Type B1:

Posterior subluxation (>50%) without significant erosion.

Narrowed posterior joint space with subchondral sclerosis or osteophytes.

Confirm if Point B has a disproportionately larger gap to the humeral head than Point C.

Type B2:

Posterior subluxation (>50%) with biconcave glenoid morphology due to posterior erosion.

Confirm biconcavity by identifying an additional concavity at an intermediate point (E) between Points B and C (see bottom-left image in reference diagram).

If no biconcavity is present, it cannot be Type B2.

Type B3:

Monoconcave morphology with severe retroversion (≥15°) or posterior subluxation (≥70%), or both.

Absence of measurable paleoglenoid differentiates it from Type B2.

Type C:

Retroversion >25°, originating from dysplasia rather than erosion.

Type D:

Anteversion or anterior subluxation (<40%).

Key Clarifications for Misclassification Prevention

For Type A2 vs. Type B2:

Central wear = Type A2; Posterior erosion with biconcavity = Type B2.

Confirm transection points for Type A2; absence of biconcavity excludes Type B2.

For Type B3 vs. Type C:

Retroversion caused by erosion = Type B3; Dysplastic retroversion (>25°) = Type C.

Reference Diagram Annotations

The attached diagrams include labeled Points A, B, C, and E, as well as key lines and angles for classification:

Concentric Glenoids: Top-right and bottom-right images show examples of Type A1 and A2 classifications.

Note how transection points distinguish A1 from A2.

Eccentric Glenoids: Bottom-left image highlights biconcavity with an E point for Type B2 classification, while top-left image shows monoconcave morphology typical of Type B3.

Do you understand these instructions"

Example 2:

Please use the attached screenshot for a reference on how to visually interpret Glenoid images.

Step 1 is to construct the scapular line using the scapular axis. You will do this by forming a straight line that originates from point A (medial scapular point) of the glenoid, and goes through the glenoid at the middle point on the glenoid itself between the glenoid points B and C, and will continue laterally throughout the humeral head (highlighted in pink). The B/C midpoint should not be located on the line directly connecting points B and C as shown in green. Forming this line is important because the head subluxation is measured relative to this line. Continue the line laterally as shown by the blue line in the diagram, and identify the posterior subluxation as the region in the diagram highlighted in blue.

Step 2 is to evaluate for a type D glenoid by seeing if the posterior subluxation is less than 50%; if yes then confirm if there is a disproportionately larger gap between point C and the glenoid head compared to point B and the glenoid head. If both of these are true then this is a type D glenoid.

If the posterior subluxation is not less than 50%, then proceed to Step 3

Step 3 is to evaluate if the glenoid is concentric or eccentric. This can be seen in the screenshot as the acute angle between the green and the blue line. This step is important because Type A glenoids MUST be concentric, and all other glenoid types MUST be eccentric. The steps can be summarized as follows:

a. Draw the B-C line (anterior to posterior rim), as demonstrated by the green lines

b. Measure the ACUTE (anterior-lateral) angle where this green line intersects the blue scapular line

c. Classification threshold:

- If angle >75° = CONCENTRIC

- If angle <75° = ECCENTRIC

Step 4 is to subsequently identify the glenoid type. The criteria is detailed in the following instructions:

4a. For glenoids identified as CONCENTRIC, separate them based on the following criteria:

Type A is a well-centered humeral head with symmetric erosion without posterior subluxation of the humeral head. Double check by confirming if the concentricity angle is close to 90 degrees.

- A1 has minor central wear or erosion in which a line drawn (shown as the green line) from the anterior (point B) to posterior (point C) rims of the native glenoid will not transect, or is tangential, to the humeral head surface as illustrated in pink. As example of this can be seen in the top-right image of the reference screenshot.

- A2 alternatively has severe or major central wear or erosion, which means that a line (shown as the green line) drawn from the anterior (point B) to posterior (point C) rims of the native glenoid will transect the humeral head past the humeral head surface as illustrated in pink. Double check A2 classification by confirming the B-C line transects the humeral head at both an anterior and posterior transection point. An example of this is the bottom-right image of the reference screenshot; the transection is indicated by the words "Transects Humeral Head" pointing to a red line on the screenshot, in addition to there being both anterior and posterior transection points.

4b. For glenoids identified as ECCENTRIC, separate them based on the following criteria:

Type B is characterized by asymmetric arthritis with posterior subluxation of the humeral head, typically >80% of the humeral head.

- Type B1 has no obvious glenoid erosion with posterior joint space narrowing, subchondral sclerosis, and osteophytes. Confirm if there is a disproportionately larger gap between point B and the glenoid head compared to point C and the glenoid head. If this is true then it is likely type B1.

- Type B2 MUST demonstrate a biconcave appearance between the B & C points due to apparent or obvious erosion of the posterior glenoid. If this line is not biconcave then it CANNOT be type B2. Confirm if there is a disproportionately larger gap between point B and the humeral head surface compared to point C and the humeral head surface. If this is true then it is likely type B2.

- Type B3 is monoconcave and posteriorly worn, with at least 15° of retroversion or at least 70% posterior humeral head subluxation, or both. The B3 glenoid with posterior subluxation without significant retroversion differs from the B1 by the presence of posterior wear.

A translational error from original reports defines the C glenoid as one with 25° of retroversion “regardless of erosion,” which has caused some misuse of that categorization; a more precise definition of the C glenoid would be one of 25° of retroversion “not caused by erosion.” This has caused erroneous labelling of B2 glenoids as C glenoids despite the presence of biconcavity or the absence of dysplasia, or both. In conclusion the C glenoid to be a dysplastic glenoid with at least 25° of retroversion “not caused by erosion.”

Define the D glenoid as one with any level of glenoid anteversion or with humeral head subluxation of less than 40% (ie, anterior subluxation).

Do you understand these instructions”

Example 3:

Please use the following criteria to analyze the unlabeled image below. Finish your reply by stating what classification of Walch Glenoid best matches the unlabeled image. For reference there is also a separate screenshot attached to this query to supplement the instructions below.

## Step 1: Construct the Scapular Line

- Identify points A, B, and C

- Draw the scapular line (pink)

- Extend the line laterally (blue)

- Identify posterior subluxation

## Step 2: Evaluate for Type D Glenoid

- Check posterior subluxation (<50%)

- Compare gaps between points B, C, and humeral head

## Step 3: Assess Concentricity

- Draw B-C line (green)

- Measure acute angle with scapular line

- Classify as concentric (>75°) or eccentric (<75°)

## Step 4: Identify Glenoid Type

### Concentric Glenoids (Type A) A1 Classification: - Draw B-C line (green) - If line ONLY touches or doesn't touch humeral head = A1 - Key visual: Line is tangential or separate from pink humeral head outline

A2 Classification: - Draw B-C line (green) - If line cuts through humeral head TWICE = A2 - Key visual: Line creates entry and exit points through pink humeral head outline

### Eccentric Glenoids

- Type B: Posterior subluxation (>80%)

- B1: No obvious erosion

- B2: Biconcave appearance

- B3: Monoconcave, posteriorly worn

- Type C: Dysplastic with ≥25° retroversion

- Type D: Anteversion or <40% subluxation

## Step 5: Review and Confirm

- Double-check classification criteria

- Ensure consistency with image features

Example 4:

Please use the attached screenshot for a reference on how to visually interpret Glenoid images.

Step 1 is to construct the scapular line using the scapular axis. You will do this by forming a straight line that originates from point A (medial scapular point) of the glenoid, and goes through the glenoid at the middle point on the glenoid itself between the glenoid points B and C, and will continue laterally throughout the humeral head (highlighted in pink). The B/C midpoint should not be located on the line directly connecting points B and C as shown in green. Forming this line is important because the head subluxation is measured relative to this line. Continue the line laterally as shown by the blue line in the diagram, and identify the posterior subluxation as the region in the diagram highlighted in blue.

Step 2 is to evaluate for a type D glenoid by seeing if the posterior subluxation is less than 50%; if yes then confirm if there is a disproportionately larger gap between point C and the glenoid head compared to point B and the glenoid head. If both of these are true then this is a type D glenoid.

If the posterior subluxation is not less than 50%, then proceed to Step 3

Step 3 is to evaluate if the glenoid is concentric or eccentric. This can be seen in the screenshot as the acute angle between the green and the blue line. This step is important because Type A glenoids MUST be concentric, and all other glenoid types MUST be eccentric. The steps can be summarized as follows:

a. Draw the B-C line (anterior to posterior rim), as demonstrated by the green lines

b. Measure the ACUTE (anterior-lateral) angle where this green line intersects the blue scapular line

c. Classification threshold:

- If angle >75° = CONCENTRIC

- If angle <75° = ECCENTRIC

Step 4 is to subsequently identify the glenoid type. The criteria is detailed in the following instructions:

4a. For glenoids identified as CONCENTRIC, separate them based on the following criteria:

Type A is a well-centered humeral head with symmetric erosion without posterior subluxation of the humeral head. Double check by confirming if the concentricity angle is close to 90 degrees.

- A1 has minor central wear or erosion in which a line drawn (shown as the green line) from the anterior (point B) to posterior (point C) rims of the native glenoid will not transect, or is tangential, to the humeral head surface as illustrated in pink. As example of this can be seen in the top-right image of the reference screenshot.

- A2 alternatively has severe or major central wear or erosion, which means that a line (shown as the green line) drawn from the anterior (point B) to posterior (point C) rims of the native glenoid will transect the humeral head past the humeral head surface as illustrated in pink. Double check A2 classification by confirming the B-C line transects the humeral head at both an anterior and posterior transection point. An example of this is the bottom-right image of the reference screenshot; the transection is indicated by the words "Transects Humeral Head" pointing to a red line on the screenshot, in addition to there being both anterior and posterior transection points.

ß

For glenoids identified as ECCENTRIC, separate them based on the following criteria:

Type B is characterized by asymmetric arthritis with posterior subluxation of the humeral head, typically >80% of the humeral head.

- Type B1 has no obvious glenoid erosion with posterior joint space narrowing, subchondral sclerosis, and osteophytes. Confirm if there is a disproportionately larger gap between point B and the glenoid head compared to point C and the glenoid head. If this is true then it is likely type B1.

- Type B2 MUST demonstrate a biconcave appearance between the B & C points due to apparent or obvious erosion of the posterior glenoid. If this line is not biconcave then it CANNOT be type B2. Confirm if there is a disproportionately larger gap between point B and the humeral head surface compared to point C and the humeral head surface. If this is true then it is likely type B2.

- Type B3 is monoconcave and posteriorly worn, with at least 15° of retroversion or at least 70% posterior humeral head subluxation, or both. The B3 glenoid with posterior subluxation without significant retroversion differs from the B1 by the presence of posterior wear.

A translational error from original reports defines the C glenoid as one with 25° of retroversion “regardless of erosion,” which has caused some misuse of that categorization; a more precise definition of the C glenoid would be one of 25° of retroversion “not caused by erosion.” This has caused erroneous labelling of B2 glenoids as C glenoids despite the presence of biconcavity or the absence of dysplasia, or both. In conclusion the C glenoid to be a dysplastic glenoid with at least 25° of retroversion “not caused by erosion.”

Define the D glenoid as one with any level of glenoid anteversion or with humeral head subluxation of less than 40% (ie, anterior subluxation).

Do you understand these instructions

Example 5:

Instructions for Glenoid Image Analysis:

Please use the attached reference diagrams to visually interpret glenoid images. Follow the steps below to classify the glenoid type accurately.

Step 1: Construct the Scapular Line

Purpose: Establish a reference line for measuring humeral head subluxation and glenoid version.

Procedure: Identify Point A (medial scapular border) and draw a straight line through the midpoint of the glenoid (between Points B and C) extending laterally.

The midpoint should represent the geometric center of the glenoid and must not lie directly on the B-C line.

Extend this line laterally through the humeral head to assess posterior subluxation (highlighted in blue in the diagrams).

Use this line as the baseline for subsequent measurements.

Step 2: Evaluate Humeral Head Subluxation

Purpose: Determine whether posterior subluxation is <50% or >50% to differentiate Type D glenoids.

Criteria: If posterior subluxation is <50%, confirm: There is a disproportionately larger gap between Point C (posterior rim) and the humeral head compared to Point B (anterior rim).

If both conditions are true, classify as Type D.

If posterior subluxation is ≥50%, proceed to Step 3.

Step 3: Assess Glenoid Concentricity

Purpose: Differentiate concentric from eccentric glenoids, a critical step in classification.

Procedure: Draw the B-C line (anterior-to-posterior rim, shown in green). Measure the acute angle where this green line intersects the scapular line (blue).

Thresholds:

Angle >75° = Concentric

Angle <75° = Eccentric

Step 4: Classify Glenoid Type

A. Concentric Glenoids

Associated only with Type A classification.

Type A1:

Minor central wear or erosion.

The B-C line does not transect or is tangential to the humeral head surface.

Example: Top-right image in reference diagram.

Type A2:

Severe central wear or erosion.

The B-C line transects the humeral head at both anterior and posterior points (as shown in red in bottom-right image of reference diagram).

Confirm by identifying both anterior and posterior transection points.

B. Eccentric Glenoids

Associated with Types B, C, or D classifications.

Type B1:

Posterior subluxation (>50%) without significant erosion.

Narrowed posterior joint space with subchondral sclerosis or osteophytes.

Confirm if Point B has a disproportionately larger gap to the humeral head than Point C.

Type B2:

Posterior subluxation (>50%) with biconcave glenoid morphology due to posterior erosion.

Confirm biconcavity by identifying an additional concavity at an intermediate point (E) between Points B and C (see bottom-left image in reference diagram).

If no biconcavity is present, it cannot be Type B2.

Type B3:

Monoconcave morphology with severe retroversion (≥15°) or posterior subluxation (≥70%), or both.

Absence of measurable paleoglenoid differentiates it from Type B2.

Type C:

Retroversion >25°, originating from dysplasia rather than erosion.

Type D:

Anteversion or anterior subluxation (<40%).

Key Clarifications for Misclassification Prevention

For Type A2 vs. Type B2:

Central wear = Type A2; Posterior erosion with biconcavity = Type B2.

Confirm transection points for Type A2; absence of biconcavity excludes Type B2.

For Type B3 vs. Type C:

Retroversion caused by erosion = Type B3; Dysplastic retroversion (>25°) = Type C.

Reference Diagram Annotations

The attached diagrams include labeled Points A, B, C, and E, as well as key lines and angles for classification:

Concentric Glenoids: Top-right and bottom-right images show examples of Type A1 and A2 classifications.

Note how transection points distinguish A1 from A2.

Eccentric Glenoids: Bottom-left image highlights biconcavity with an E point for Type B2 classification, while top-left image shows monoconcave morphology typical of Type B3.

Do you understand these instructions"
